# Perioperative management of a pediatric patient with suspected type 1 von Willebrand disease undergoing tonsillectomy: a case report

**DOI:** 10.1186/s40981-019-0276-4

**Published:** 2019-08-27

**Authors:** Hiroyuki Oshika, Yukihide Koyama, Koichi Tsuzaki, Kohmei Ida, Tomio Andoh

**Affiliations:** 1 Department of Anesthesiology, Mizonokuchi Hospital, Teikyo University School of Medicine, Kawasaki, Japan; 2 Department of Anesthesia, Nippon Koukan Hospital, 1-2-1 Koukan-dori, Kawasaki-ku, Kawasaki-shi, Kawasaki, Kanagawa Prefecture 210-0852 Japan; 3 Department of Pediatrics, Mizonokuchi Hospital, Teikyo University School of Medicine, Kawasaki, Japan

**Keywords:** Confact F^®^, Plasma-derived factor VIII concentrate, von Willebrand disease, von Willebrand factor, VWD, VWF

## Abstract

**Background:**

Von Willebrand disease (VWD) is the most common inherited bleeding disorder in humans. Coagulopathies such as VWD are evidently risk factors for post-surgical bleeding. Perioperative management of patients with VWD remains controversial and is a major clinical concern.

**Case presentation:**

A 5-year-old girl was scheduled for tonsillectomy under general anesthesia. Preoperative laboratory tests revealed prolongation of activated partial thromboplastin time and a mild decrease in von Willebrand factor (VWF) activity. Prophylactic administration of desmopressin or VWF was not performed. During tonsillectomy, oozing from the surgical wound was uncontrollable by conventional hemostasis techniques, but complete hemostasis was ensured by plasma-derived coagulation factor VIII concentrate containing VWF.

**Conclusion:**

Pediatric patients with mild abnormalities in preoperative laboratory tests may have coagulopathies. Prophylactic intervention and/or the preparation of a sufficient amount of coagulation factor VIII concentrate containing VWF may be required in patients suspected of having VWD or with mild VWF deficiency.

## Background

Von Willebrand disease (VWD) is the most common inherited bleeding disorder in humans, with reported prevalence estimates ranging from 3 to 4 per 100,000 to as high as 1.3% of the population [1, 2]. It is caused by the deficiency or dysfunction of von Willebrand factor (VWF), a protein that mediates the initial adhesion of platelets at sites of vascular injury and also acts as a carrier protein for blood coagulation factor VIII [3]. When VWF levels are decreased or its function is affected, platelet plug formation becomes ineffective. VWD is classified into three major categories; partial quantitative deficiency (type 1), qualitative deficiency (type 2), and total deficiency (type 3).

Post-tonsillectomy hemorrhage is a common complication, with an overall frequency of approximately 5% [4]. Coagulopathies are evidently risk factors for post-tonsillectomy hemorrhage [5–8]. Accordingly, perioperative management of patients with VWD is a major clinical concern.

Herein, we describe the case of a pediatric patient who was suspected of having type 1 VWD pre-tonsillectomy, in which anesthetic management was successfully achieved via plasma-derived factor VIII concentrate containing VWF (Confact F^®^; KM Biologics Co., Ltd., Kumamoto, Japan) during the operation instead of preoperative desmopressin, which has been recommended for the prevention of postoperative bleeding in patients with type 1 VWD [9–11].

## Case presentation

A 5-year-old girl weighing 20 kg and 109 cm in height was presented with obstructive sleep apnea syndrome. She was scheduled for bilateral tonsillectomy under general anesthesia. Preoperative laboratory tests were unremarkable except prolonged activated partial thromboplastin time (APTT) of 44.0 s (normal range 25–35 s). All other preoperative laboratory test results were within normal limits. Although there was no history of episodes of bleeding diathesis in the patient or her relatives, a pediatrician was consulted with regard to her APTT prolongation. The results of further laboratory tests included blood coagulation factor XII 15.7% (normal range 50–150%), coagulation factor VIII 81.3% (normal range 70–140%), VWF activity 41% (normal range 50–100%), and VWD antigen level 60% (normal range 50–100%). Because there were no episodes of bleeding diathesis and only a mild decrease in VWF activity, she was putatively diagnosed with type 1 VWD and/or coagulation factor XII deficiency, but the diagnosis of VWD was not definite in this case. The pediatrician surmised that there was no need for prophylactic administration of desmopressin or plasma-derived coagulation factor VIII concentrate containing VWF (Confact F^®^) before surgery, but recommended the administration of Confact F^®^ immediately if hemostasis complications emerged during surgery. With regard to coagulation factor XII deficiency, no supplemental treatment was required.

In accordance with the pediatrician’s recommendations, 200 mg tranexamic acid (10 mg/kg) was administered intravenously three times the day before surgery. In the operating room, general anesthesia was induced via intravenous administration of 0.3 mg atropine sulfate, 25 μg fentanyl, 40 mg propofol, and 10 mg rocuronium, followed by tracheal intubation with a 4.5-mm cuffed oral RAE^®^ tracheal tube (Nellcor/Tyco Healthcare, Pleasanton, California, USA). Her lungs were ventilated mechanically throughout the operation. Anesthesia was maintained via 1 L/min of oxygen and 2 L/min of air with 2% sevoflurane, and intravenous fentanyl and rocuronium.

During surgery the patient’s blood pressure and heart rate were stable and her body temperature ranged from 36.3 to 36.9 °C. After bilateral tonsillectomy, oozing from the surgical wound did not stop despite conventional bipolar coagulation, suture ligature, and astriction with gauze packing on the surgical wound. Thus, in accordance with the pediatrician’s recommendation, 850 units of Confact F^®^ including 850 units of coagulation factor VIII and 2040 units of VWF was administered immediately. Complete hemostasis was achieved after administration of 850 units of Confact F^®^. The recommended initial dose of Confact F^®^ was 40–50 units/kg in the present case. At the end of the surgery, 200 mg sugammadex was administered, then tracheal extubation was gently performed. Anesthesia time was 171 min, and the estimated amount of bleeding was 10 g. The patient exhibited no signs of pain or discomfort during emergence. No postoperative bleeding occurred, and she was discharged from hospital without any complications on postoperative day 3.

## Discussion

The diagnosis of VWD is based on a history of bleeding diathesis, often with a family history of bleeding symptoms or diagnosed VWD, and confirmatory laboratory tests [3]. VWD diagnosis and management remain challenging however, especially in children and adolescents with no history of bleeding diathesis. Such patients have usually not experienced significant hemostatic challenges and thus may lack any history of bleeding diathesis [12]. Therefore, further examinations should be conducted when a pediatric patient exhibits abnormalities in preoperative laboratory tests even in the absence of any history of abnormal bleeding to investigate whether the patient has any coagulopathies.

In general, preoperative management in patients with VWD may include desmopressin, coagulation factor VIII concentrates containing VWF, cryoprecipitate, and topically applied hemostatic agents [11]. Desmopressin has been shown to stimulate the release of VWF and coagulation factor VIII [10]. Consequently, preoperative desmopressin administration may effectively reduce the risk of postoperative bleeding in pediatric patients with VWD undergoing adenotonsillectomy [9]. However, its use is not without risk. Fluid and electrolyte management is required to prevent hyponatremia with possible seizures and fluid retention [9]. There are also potential risks associated with factor concentrates and cryoprecipitate, including allergic reactions, thrombotic complications, and the transmission of blood-borne pathogens. Thus, to date, it remains unclear what preoperative management in patients with VWD is most appropriate for preventing postoperative bleeding.

In clinical practice, the preoperative management of patients with VWD currently depends on the type of VWD and the patient’s condition. The present patient was not definitely diagnosed with VWD, but type 1 VWD was suspected because VWF deficiency was mild and neither she nor her relatives had any history of episodes of bleeding diathesis. Consequently, the pediatrician did not recommend prophylactic administration of Confact F^®^ or desmopressin before surgery, but advised us to prepare Confact F^®^ and administer it immediately if hemostasis could not be achieved during surgery via conventional techniques. The National Heart, Lung, and Blood Institute (NHLBI) recommends that levels of VWF and coagulation factor VIII activities of at least 30%—and preferably over 50%—should be maintained for 1–5 days for minor surgery [3]. Tonsillectomy is not a major surgery, but there is a high risk of bleeding during and after the procedure [4]. Previous reports indicated that bleeding rates in children with known coagulopathies are between 2% and 17% [5–8]. Sun et al. [7] reported that the rate of delayed hemorrhage after tonsillectomy in children with VWD or hemophilia was much higher, especially in older children. Considering all the relevant factors including the patient’s condition, laboratory tests, the type of surgery, and the NHLBI recommendations [3], we followed the pediatrician’s instructions. In the current case, coagulation factor VIII concentrate containing VWF was very effective for achieving hemostasis during surgery. Notably, however, in some previously reported cases, it was evidently ineffective in patients with VWD [13, 14]. The initial dose of factor VIII concentrate containing VWF may be insufficient in some cases due to the difference of the type of VWD or the severity of VWF deficiency. Thus, if uncontrollable bleeding occurs in similar cases during surgery, further administration of factor VIII concentrate containing VWF should be considered. Moreover, further interventions should be considered such as cryoprecipitate, topically applied hemostatic agents, and the newly developed recombinant VWD product that has recently become available [12].

In conclusion, even slight abnormalities in preoperative laboratory tests should not be overlooked in pediatric patients even in the absence of any history of bleeding diathesis. Such pediatric patients may have coagulopathies that have not previously come to light because they have not experienced any substantial hemostatic challenges and thus have no history of abnormal bleeding. There may be a need to administer preoperative prophylactic intervention and/or prepare a sufficient amount of coagulation factor VIII concentrate containing VWF in cases involving patients suspected of having VWD or with mild deficiency of VWF undergoing minor surgeries with a substantial risk of bleeding such as adenotonsillectomy.

## Data Availability

The data are not available for public access because of patient privacy concerns, but are available from the corresponding author on reasonable request.

## Abbreviations

APTT: Activated partial thromboplastin time
NHLBI: National Heart, Lung, and Blood Institute
VWD: von Willebrand disease
VWF: von Willebrand factor
